# A novel risk signature based on liquid-liquid phase separation-related genes reveals prognostic and tumour microenvironmental features in clear cell renal cell carcinoma

**DOI:** 10.18632/aging.205691

**Published:** 2024-03-27

**Authors:** Qing Lu, Ping Xi, Suling Xu, Zhicheng Zhang, Binbin Gong, Ji Liu, Qiqi Zhu, Ting Sun, Shaoxing Zhu, Ru Chen

**Affiliations:** 1 Department of Urology, Fujian Medical University Union Hospital, Fuzhou 350001, Fujian, P.R. China; 2 Department of Urology, The First Affiliated Hospital of Nanchang University, Nanchang 330006, Jiangxi, China; 3 Department of Surgery, Fuzhou First People’s Hospital, Fuzhou 344000, Jiangxi, China

**Keywords:** clear cell renal cell carcinoma, liquid-liquid phase separation, risk signature, tumour microenvironment, prognostic

## Abstract

Background: Clear cell renal cell carcinoma(ccRCC) is one of the most common malignancies. However, there are still many barriers to its underlying causes, early diagnostic techniques and therapeutic approaches.

Materials and Methods: The Cancer Genome Atlas (TCGA)- Kidney renal clear cell (KIRC) cohort differentially analysed liquid-liquid phase separation (LLPS)-related genes from the DrLLPS website. Univariate and multivariate Cox regression analyses and LASSO regression analyses were used to construct prognostic models. The E-MTAB-1980 cohort was used for external validation. Then, potential functions, immune infiltration analysis, and mutational landscapes were analysed for the high-risk and low-risk groups. Finally, quantitative real-time polymerase chain reaction (qRT-PCR) experiments as well as single-cell analyses validated the genes key to the model.

Results: We screened 174 LLPS-related genes in ccRCC and constructed a risk signature consisting of five genes (CLIC5, MXD3, NUF2, PABPC1L, PLK1). The high-risk group was found to be associated with worse prognosis in different subgroups. A nomogram constructed by combining age and tumour stage had a strong predictive power for the prognosis of ccRCC patients. In addition, there were differences in pathway enrichment, immune cell infiltration, and mutational landscapes between the two groups. The results of qRT-PCR in renal cancer cell lines and renal cancer tissues were consistent with the biosignature prediction. Three single-cell data of GSE159115, GSE139555, and GSE121636 were analysed for differences in the presence of these five genes in different cells.

Conclusions: We developed a risk signature constructed based on the five LLPS-related genes and can have a high ability to predict the prognosis of ccRCC patients, further providing a strong support for clinical decision-making.

## INTRODUCTION

Kidney cancer, medically termed renal cell carcinoma, emerges as a malignant tumor that originates within the kidney tissue. Its prevalence is on a consistent rise, positioning it as one of the frequently encountered malignancies on a global scale [1]. Complex heterogeneity is evident at both the clinical and molecular levels, as various subtypes exhibit notable distinctions in terms of biological characteristics, molecular mechanisms, and responses to therapeutic interventions [2]. Clear cell renal cell carcinoma stands out as one of the prevalent forms of kidney cancer, encompassing the majority of cases within this category [3]. Emerging from the clear cells within the kidney, typically located in the endothelial cell lining of the renal tubules, this malignancy exhibits a propensity for heightened invasiveness and mobility. It is usually highly invasive and migratory, resulting in high mortality and poor prognosis [4, 5]. Timely diagnosis plays a pivotal role in the effective management and prognosis of ccRCC. Nevertheless, due to the frequently elusive symptoms during its initial phases, ccRCC tends to be identified at an advanced stage, thereby curtailing the window for prompt medical intervention [6, 7]. Despite notable advancements in both clinical and fundamental research, the understanding of kidney cancer’s pathogenesis, the development of early diagnostic techniques, and the formulation of effective therapeutic approaches continue to encounter substantial challenges [8–10]. As a result, the pursuit of more precise diagnostic indicators for the early stages of renal cancer has emerged as a focal point in current research endeavors.

Cancer is an unbridled ailment characterized by the unrestrained growth of cells, orchestrated through intricate biochemical pathways that operate outside the boundaries of normal equilibrium. This equilibrium, although paradoxically termed non-homeostatic, hinges on the meticulous spatiotemporal orchestration of biochemical processes. Within eukaryotic cells, numerous organelles encased by membranes partition the cell into distinct compartments, each capable of executing specific biochemical reactions. However, coexisting with these membrane-bound entities are certain organelles lacking membranes, such as nucleoli, P granules in nematodes, Cajal vesicles, nucleosome arrays, PML nuclear bodies (NBs), foci of DNA damage, compartments related to X-chromosome inactivation (XCI), paraspeckles, ribonucleoprotein (RNP) granules, stress granules, proteasomes, autophagosomes, and synaptic vesicle motifs [11]. These membrane-less structures are primarily demarcated through the mechanism of LLPS, wherein distinct components segregate based on their liquid properties [12–14]. LLPS plays a pivotal role in an array of biological processes, encompassing the modulation of nuclear operations, autophagy, and DNA damage response, among others [15–17]. Moreover, a mounting body of research underscores the proposition that irregularities in LLPS might intricately intertwine with the progression of cancer [18–20].

Previous studies have shown that LLPS-related gene markers can be used as prognostic markers in patients with some tumors, such as prostate cancer, endometrial cancer, breast cancer, hepatocellular carcinoma, and epithelial ovarian cancer [21–25]. Consequently, the primary objective of this investigation was to develop and substantiate a predictive model for prognosticating LLPS- related genes within ccRCC. Subsequent to this, we delved into the plausible interrelation between the refined model and immune infiltration as well as mutations. To conclude, we executed PCR validation and conducted single-cell analysis on the genes identified through screening.

## RESULTS

### Construction and validation of a prognostic prediction model for LLPS-related genes in ccRCC

**Figure 1:**
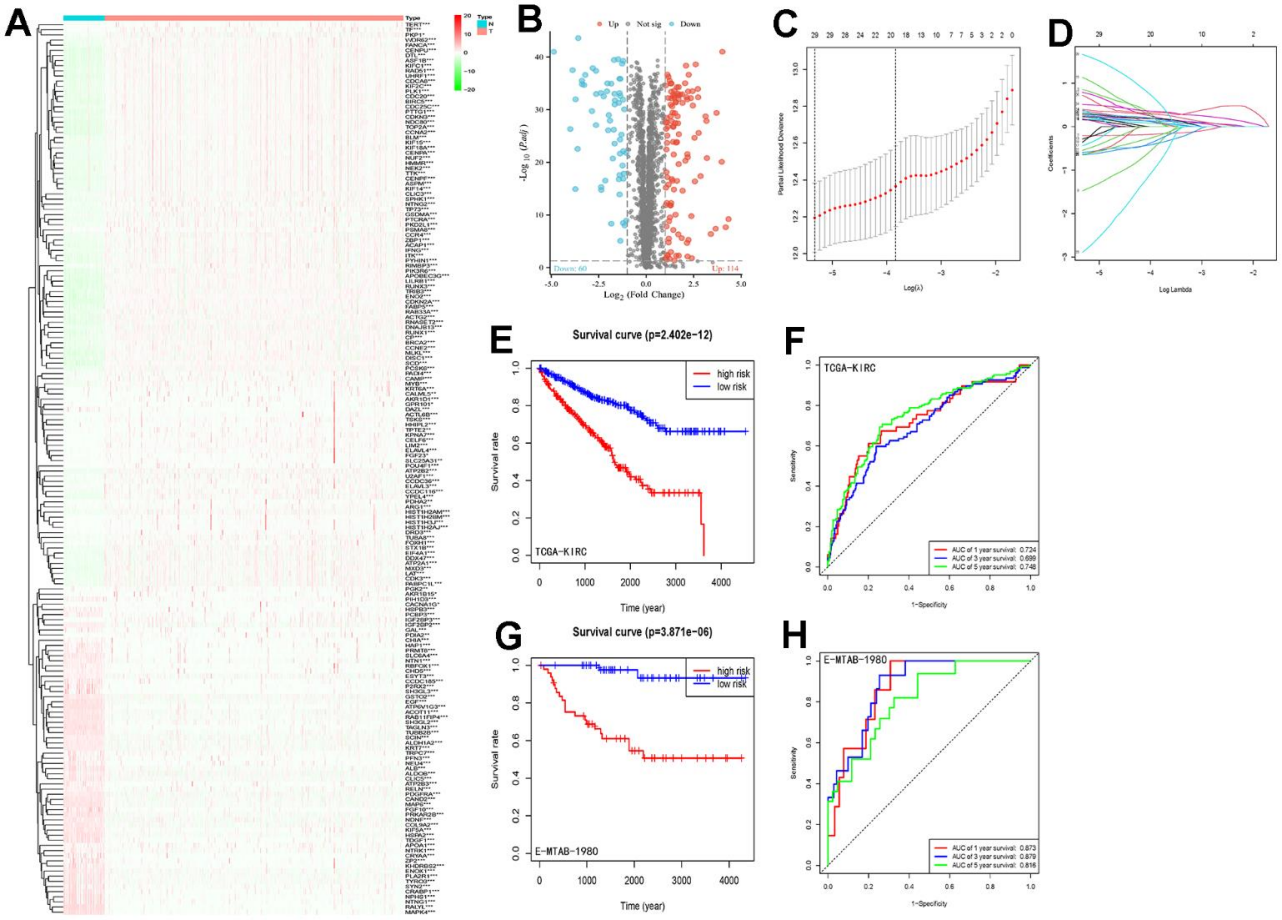
**LLPS differential gene screening and risk signature establishment and assessment.** (**A**) Heatmap demonstrating the expression of LLPS-related genes in the TCGA-KIRC cohort; (**B**) Volcano plot demonstrating the LLPS-based expression in the TCGA-KIRC cohort, containing 60 down-regulated genes and 114 up-regulated genes; (**C**, **D**) LASSO Cox regression analysis was conducted to screen the key genes; (**E**) Kaplan-Meier analysis of survival differences between high-risk and low-risk groups in the TCGA-KIRC cohort; (**F**) ROC curves demonstrating the accuracy of risk-signature judgement of LLPS-related gene constructs for ccRCC patients with 1-, 3- and 5-year prognosis; (**G**) Kaplan-Meier analysis in the E-MTAB-1980 cohort to validate the survival difference between the high-risk and low-risk groups distinguished by this risk signature; (**H**) ROC curve to validate the ability of this signature to judge prognosis in the E-MTAB-1980 cohort. (*p < 0.05, **p < 0.01, ***p < 0.001).

Differential expression analysis of 3611 LLPS-related genes from the DrLLPS database in the TCGA-KIRC cohort identified 114 up-regulated and 60 down-regulated genes (Figure 1A, 1B). By univariate Cox regression analysis, we identified 137 prognostic LLPS-related genes associated with overall survival (Supplementary Table 2). Based on this result, the λ-optimal value of LASSO Cox regression analysis incorporated five genes (CLIC5, MXD3, NUF2, PABPC1L, and PLK1) to construct a risk signature characterization of LLPS-related genes (Figure 1C, 1D). The risk score for each patient was calculated according to the following formula: Risk score = 0.162*NUF2 expression + 0.317*PLK1 expression + 0.107*MXD3 expression + 0.111*PABPC1L expression + (-0.049)*CICL5 expression. The median risk score values were used to categorize patients into high-risk and low-risk groups. And Kaplan-Meier analysis revealed that the higher the risk scores of ccRCC patients, the lower the overall survival rate of patients (p=2.402e-12) (Figure 1E). To further validate the accuracy of the model’s prediction, the receiver operating characteristic (ROC) curve results showed that the model had a strong ability to predict the survival rates of ccRCC patients at 1, 3, and 5 years (corresponding to area under the curve (AUC) values of 0.724, 0.699, and 0.748, respectively) (Figure 1F).

We scored the risk score prediction model in the validation set E-MTAB-1980 cohort similarly for each patient, categorizing them into high- and low-risk groups based on the median risk score. Combined with survival time the same found that the overall survival time of patients in the high-risk group was significantly lower than that of patients in the low-risk group (Figure 1G). The ROC curve curves predicted AUC values of 0.873, 0.879, and 0.816 at 1, 3, and 5 years, respectively (Figure 1H). Results from the TCGA-KIRC and E-MTAB-1980 cohorts consistently demonstrated that our constructed LLPS-related gene risk signature has strong ability and accuracy for prognostic prediction of ccRCC patients.

### Risk scores and clinically relevant variables

**Figure 2:**
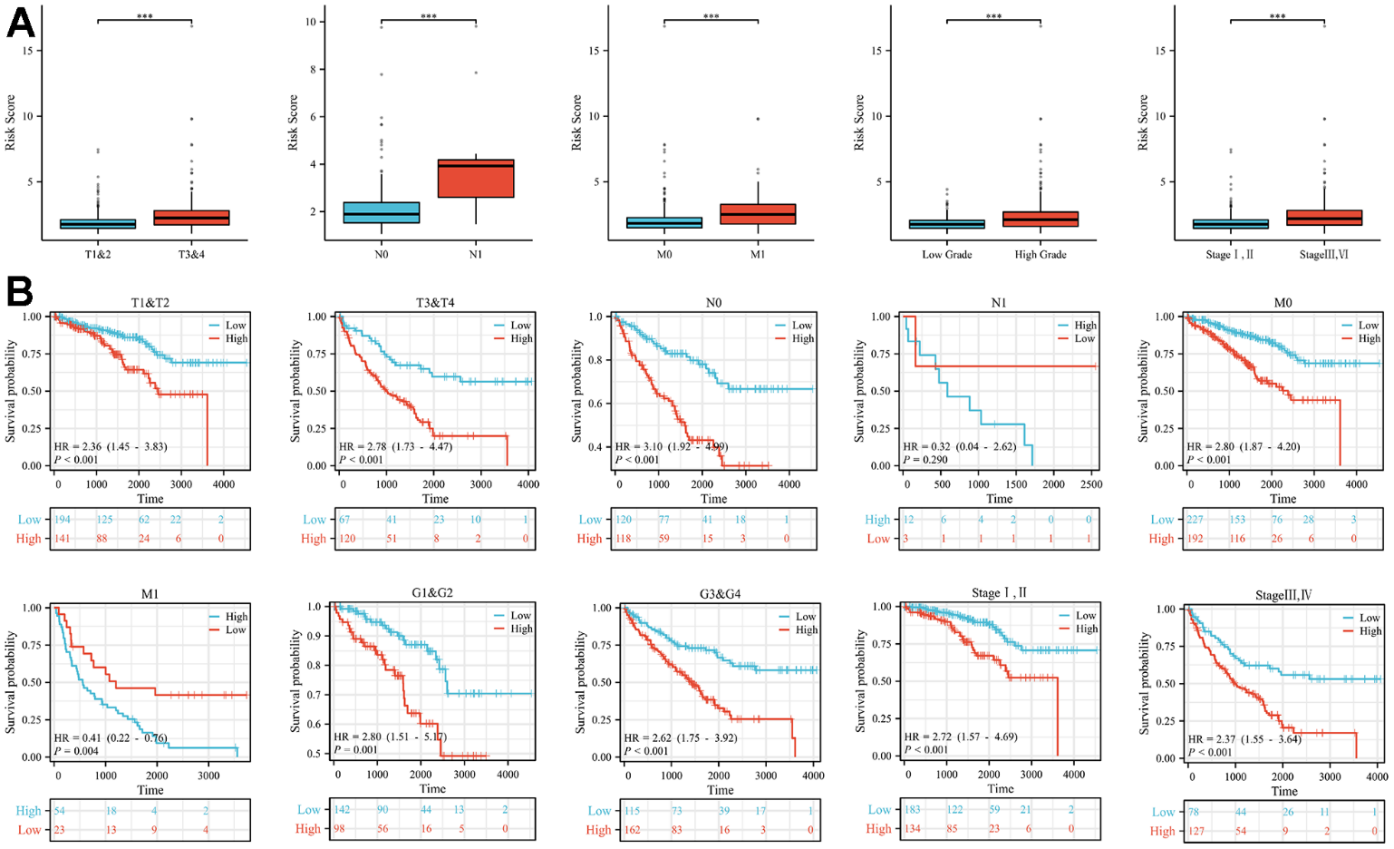
**Differences in risk scores and differences in survival in subgroups with different clinical characteristics.** (**A**) In subgroups with different clinical characteristics (T stage, N stage, M stage, tumour grade and tumour stage), the higher the malignancy of the disease, the higher the risk score. (**B**) The prognosis of patients in the high-risk group in the subgroups with different clinical features was significantly worse than that in the low-risk group, except for the N1 subgroup (***p < 0.001).

Patients were categorized into different subgroups according to their T, N, M, clinical and pathological stages. The higher the degree of malignancy, the higher the risk score (Figure 2A). And the Kaplan-Meier analysis showed that the overall survival rate of the high-risk group was statistically significantly lower than that of the low-risk group in the different clinical subgroups, with the difference being statistically significant except for patients in the N1 group, where no statistical difference was observed (Figure 2B).

### Development and validation of a nomogram combining age, tumor stage, and risk score

**Figure 3:**
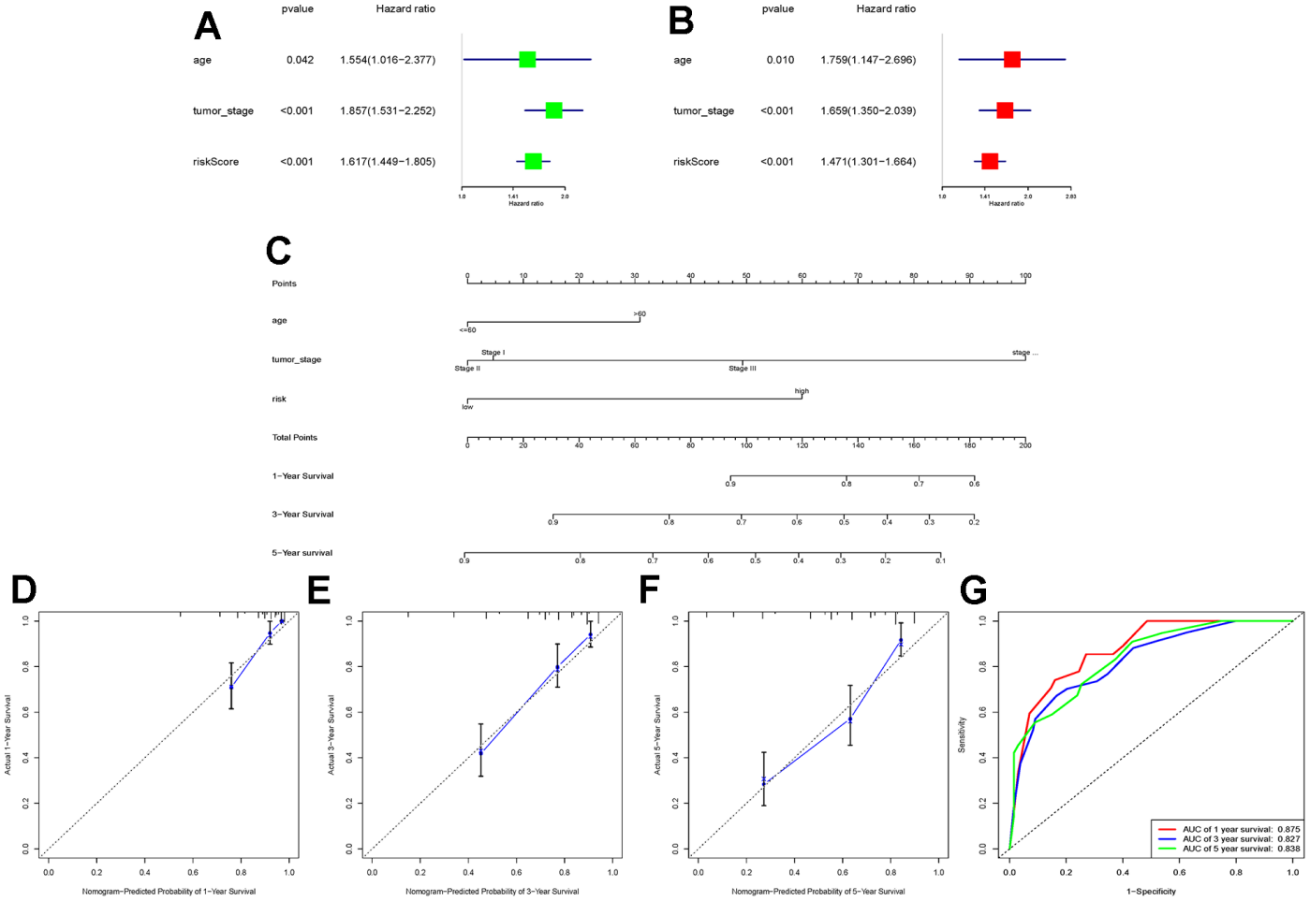
**Construction of a novel nomogram based on the TCGA-KIRC cohort.** (**A**, **B**) Combining age and tumour stage, univariate and multivariate Cox regression analyses were performed to determine the risk score as an independent risk factor for ccRCC patients. (**C**) A nomogram combining age, tumour stage, and risk score was constructed; (**D**–**F**) Calibration charts at 1, 3, and 5 years were plotted to assess the accuracy of the nomogram; (**G**) ROC curves were used to further validate the ability of the nomogram to assess the patients’ prognosis at 1, 3, and 5 years.

**Figure 4:**
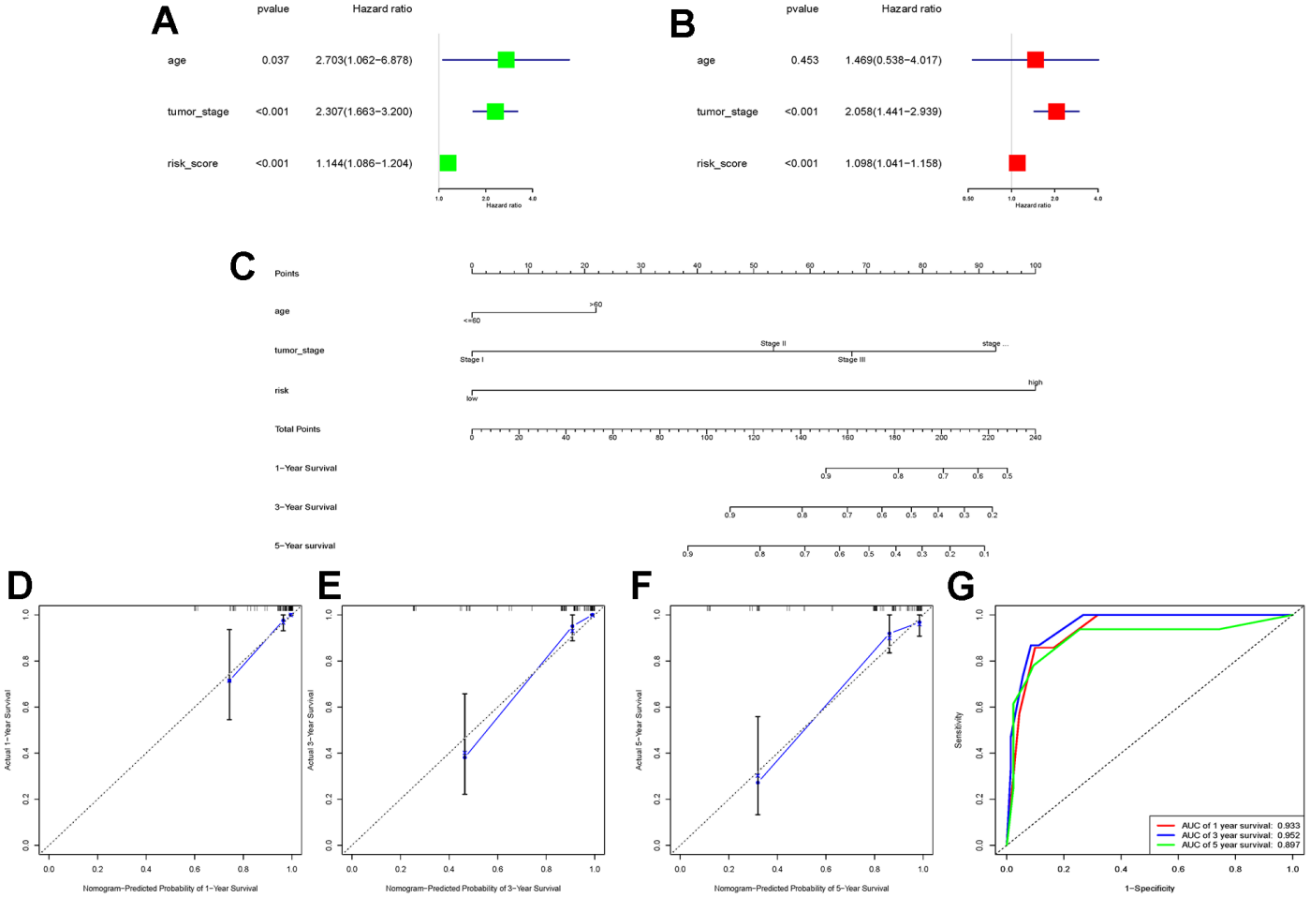
**E-MTAB-1980 cohort data validation of the constructed nomogram.** (**A**, **B**) Univariate and multivariate Cox regression analyses were performed in the E-MTAB-1980 cohort to validate that the risk score could be used as an independent risk factor; (**C**) The same combination of age, tumour stage, and risk score was used to construct the nomogram in the E-MTAB-1980 cohort; (**D**–**F**) Calibration charts at 1, 3, and 5 years were used to further assess the accuracy of the nomogram. to further assess the accuracy of the nomogram; (**G**) ROC curves demonstrating the ability of this nomogram to assess patients’ prognosis at 1, 3, and 5 years.

To further explore whether the risk score could serve as an independent risk factor for ccRCC patients, univariate and multivariate Cox regression analyses were performed in conjunction with age and tumor stage. The results of both TCGA-KIRC and E-MTAB-1980 cohort analyses indicated that the risk score of LLPS-related genetic features could serve as an independent predictor of prognosis for ccRCC patients (Figures 3A, 3B, 4A, 4B). Nomogram was further constructed to predict patients’ overall survival (OS) (Figures 3C, 4C). Calibration chart and C-indexes of both cohorts showed that high agreement in predicting patients’ survival at 1, 3, and 5 years with the actual survival (C-index for TCGA-KIRC cohort = 0.78 (se = 0.023); C-index for E-MTAB-1980 cohort = 0.876 (se = 0.034)) (Figures 3D–3F, 4D–4F). The reliability of this finding was further verified by the AUC values corresponding to the ROC curves (TCGA-KIRC cohort AUC values: 0.875, 0.827, 0.838; E-MTAB-1980 cohort AUC values: 0.933, 0.952, 0.897, respectively) (Figures 3G, 4G).

### Differences in potential functional and pathway enrichment analyses between patients in high- and low-risk groups

**Figure 5:**
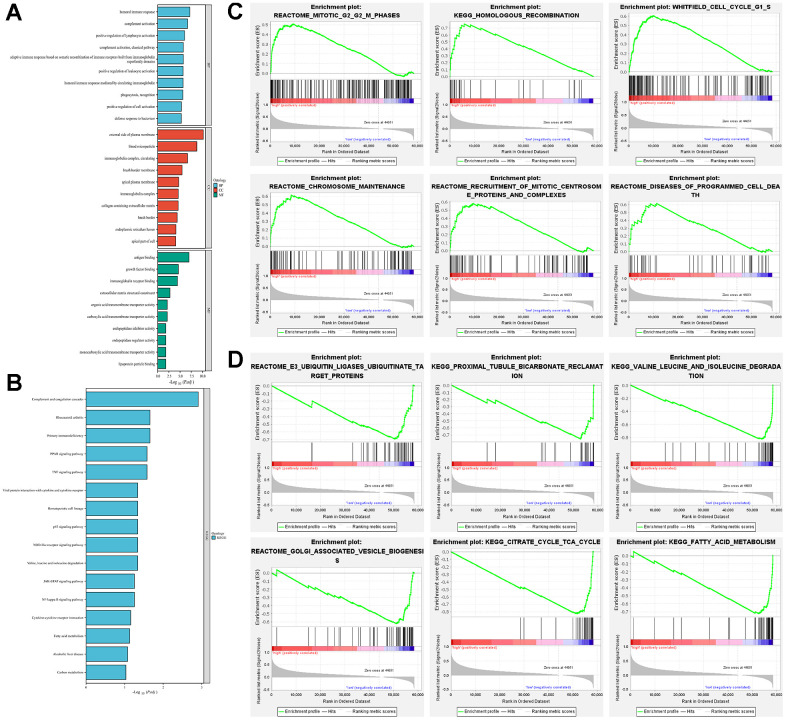
**Exploration of potential function in high-risk and low-risk groups.** (**A**) GO analysis explored the potential function in terms of BP, CC, and MF; (**B**) KEGG analysed the potential pathway enrichment; (**C**) GSEA analysed the potential pathway enrichment in the high-risk group; (**D**) GSEA analysis demonstrated the potential pathway enrichment in the low-risk group.

The results of GO and KEGG analyses indicated that the differential gene functions in the high- and low-risk groups were involved in a variety of signaling pathways. In biological processes (BP), they were mainly involved in immune-related processes, such as humoral immune response, complement activation and positive regulation of lymphocyte activation. In cellular components (CC), it is mainly enriched in processes such as external side of plasma membrane, blood microparticle, and circulating immunoglobulin complex. In terms of molecular function (MF), it mostly binds to a variety of substances (e.g., antigen, growth factor, immunoglobulin receptor, etc.) and regulates the activity of a variety of substances (e.g., organic acid transmembrane transporter, carboxylic acid transmembrane transporter, carboxylic acid transmembrane transporter, endopeptidase inhibitor, endopeptidase regulator, and monocarboxylic acid transmembrane transporter) and as structural components of the extracellular matrix (Figure 5A). Kyoto Encyclopedia of Genes and Genomes (KEGG) enrichment was shown to be involved in some classical pathways, such as complement and coagulation cascade, Primary immunodeficiency, peroxisome proliferators-activated receptor (PPAP) signaling pathway, tumor necrosis factor (TNF) signaling pathway, and p53 signaling pathway (Figure 5B). We further analyzed the signaling enrichment analysis of patients in the high-risk and low-risk groups. The high-risk group was enriched in cell cycle and mitosis related pathways (Figure 5C). While for the low-risk group was mainly enriched in substance metabolism related pathways, such as E3 ubiquitin ligase ubiquitination target proteins, proximal tubule bicarbonate recycling, valine, leucine, and isoleucine degradation, Golgi-related vesicle biogenesis, and fatty acid metabolism (Figure 5D).

### Risk score analysis of LLPS-related genes associated with immunity and mutation

**Figure 6:**
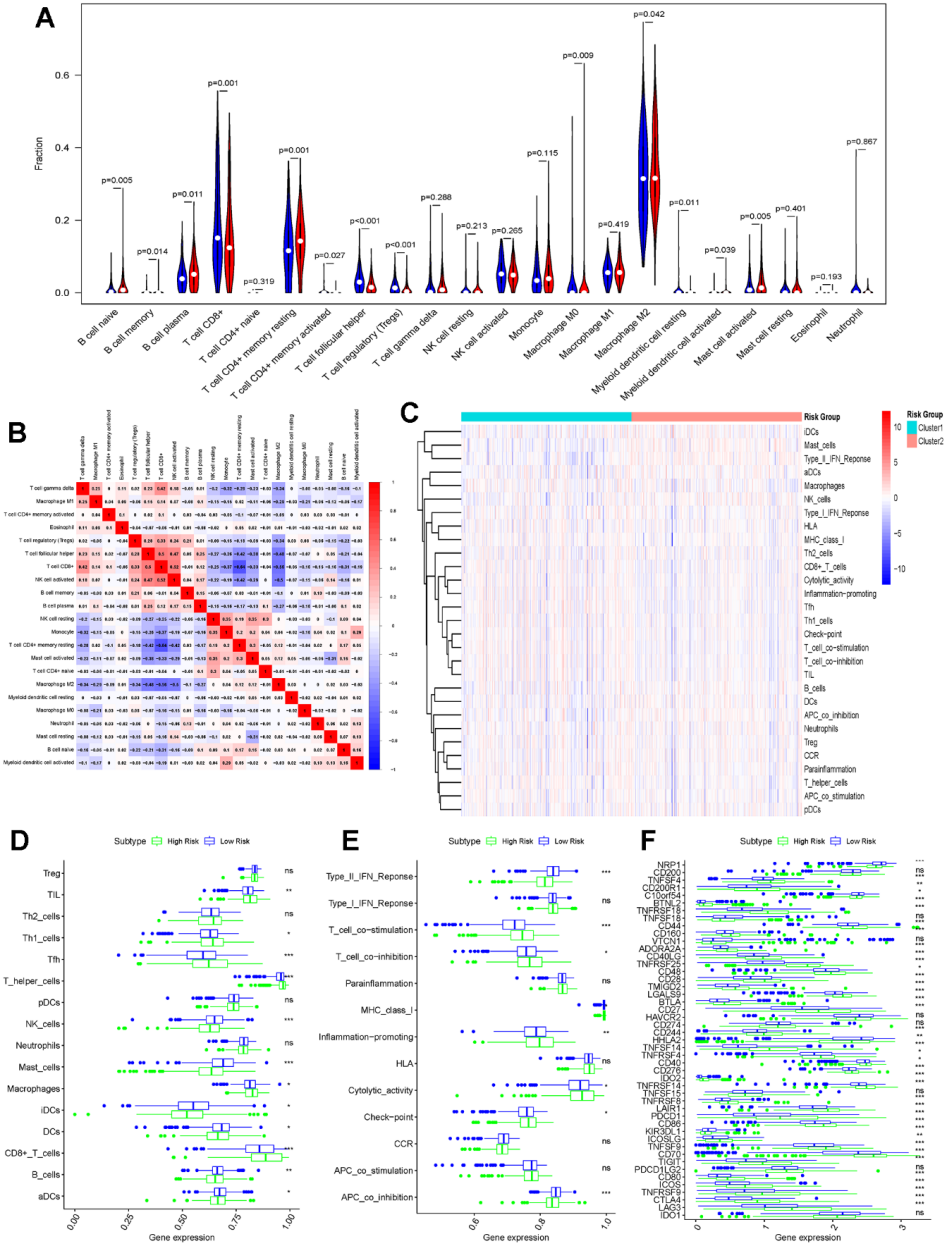
**Differences in immune function between high-risk and low-risk groups.** (**A**) CIBERSORT algorithm analysing the landscape of immune infiltration in patients in the high-risk and low-risk groups; (**B**) Immune cell correlation analysis in the CIBERSORT algorithm; (**C**) Heatmap showing the ssGESA algorithm analysing the differences in immune cells and immune infiltration in the high-risk and low-risk groups (Cluster1 represents the high-risk group; Cluster2 represents the low-risk group); (**D**) Bar graph demonstrating the difference in immune cell infiltration in the high-risk and low-risk groups under the ssGESA algorithm; (**E**) Differences in immune function between high-risk and low-risk groups by the ssGESA algorithm are demonstrated using bar graphs; (**F**) Bar graphs further demonstrating differences in the expression of immune checkpoints in the high-risk and low-risk groups. (*p < 0.05, **p < 0.01, ***p < 0.001, “ns”: not statistically significant).

Analysis of the immune infiltration landscapes of patients in the high-risk and low-risk groups by the CIBERSORT algorithm revealed that most of the immune cells (e.g., B cell naïve, B cell memory, B cell plasma, T cell CD4+ memory resting, Macrophage M0, Myeloid dendritic cell resting, Mast cell activated) infiltrated in higher abundance in the low-risk group, whereas some immune cells (e.g., T cell CD8+, T cell CD4+ memory activated, T cell follicular helper, T cell regulatory (Tregs), Macrophage M2, Myeloid dendritic cell activated) had a higher infiltration abundance in the high-risk group (Figure 6A). The correlation of these immune cells is also demonstrated in Figure 6B. Similarly in the single sample gene set enrichment analysis (ssGESA) algorithm CD8+_T cells, Macrophages, T helper cells, Tfh, Th1 cells, TIL cells had higher abundance in the high-risk group than in the low-risk group; NK cells, Mast cells, iDCs, DCs, aDCs the results were opposite (Figure 6C, 6D). In addition, the immune function of each sample was also scored, and it was not difficult to find that the scores were higher in the high-risk group in most of the immune functions, but the scores were higher in the low-risk group for antigen-presenting cell (APC) co-inhibition, Type II IFN Response (Figure 6E). In order to better verify the extent of immune response in different groups of patients, we analyzed the expression of immune checkpoints separately. Common immune checkpoints in kidney cancer (PD-1, CTLA4 and LAG3) were more significantly expressed in the high-risk group (Figure 6F).

**Figure 7:**
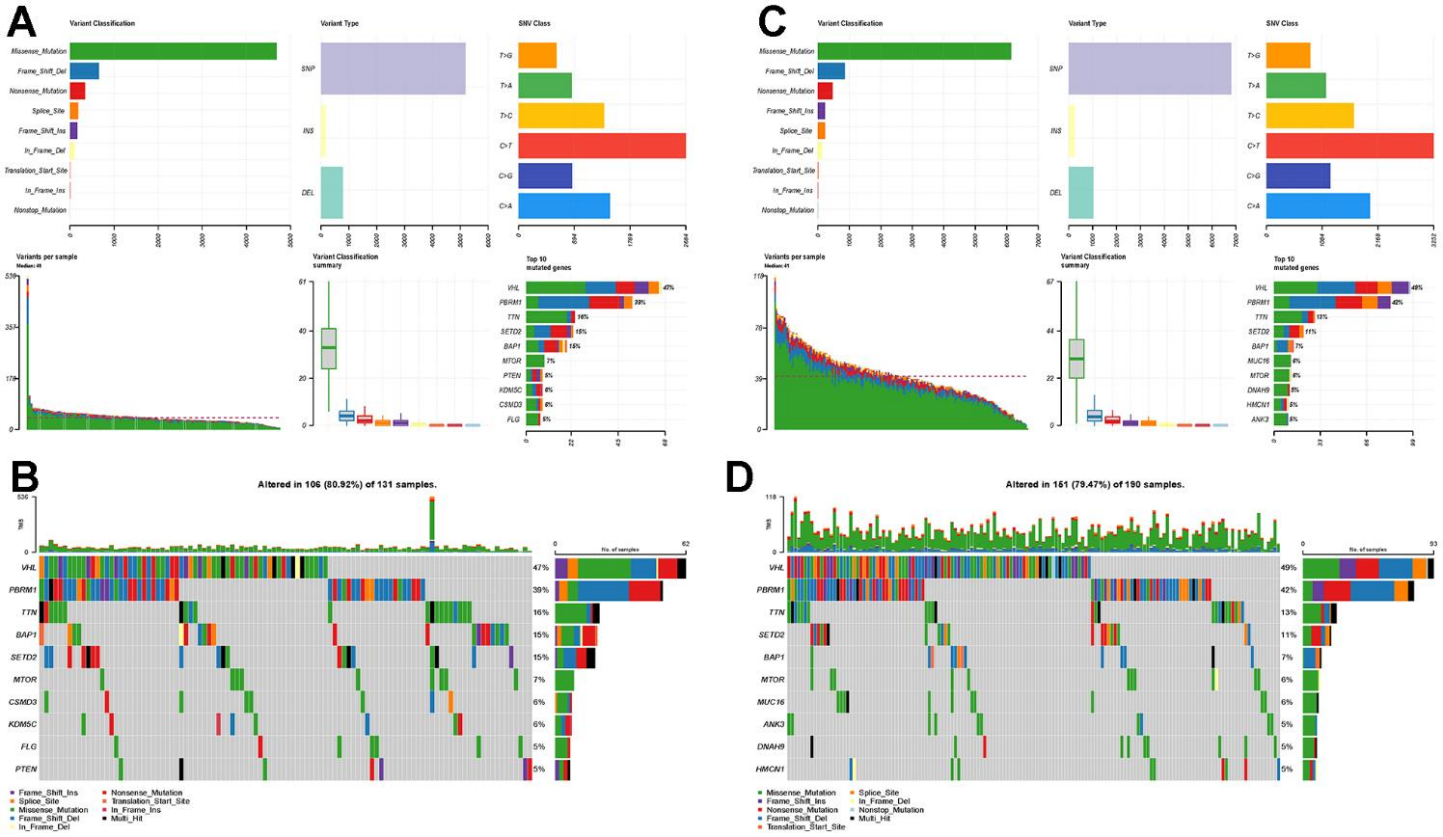
**Mutational landscapes in the high-risk and low-risk groups.** (**A**) Overall display of the differences in mutation landscapes in the high-risk group; (**B**) Waterfall plot showing the top genes with the most mutations in the high-risk group and the corresponding mutation types; (**C**) Overall mutation landscapes in the low-risk group; (**D**) Waterfall plot displaying the mutation status of the top ten genes in the low-risk group.

The high-risk and low-risk groups differed in variant classification and the 10 genes with the highest mutation frequencies, while there were no differences in variant type and SNV class (Figure 7A–7D).

### Validation and single-cell analysis of key genes in prognostic prediction models for LLPS-related genes

**Figure 8:**
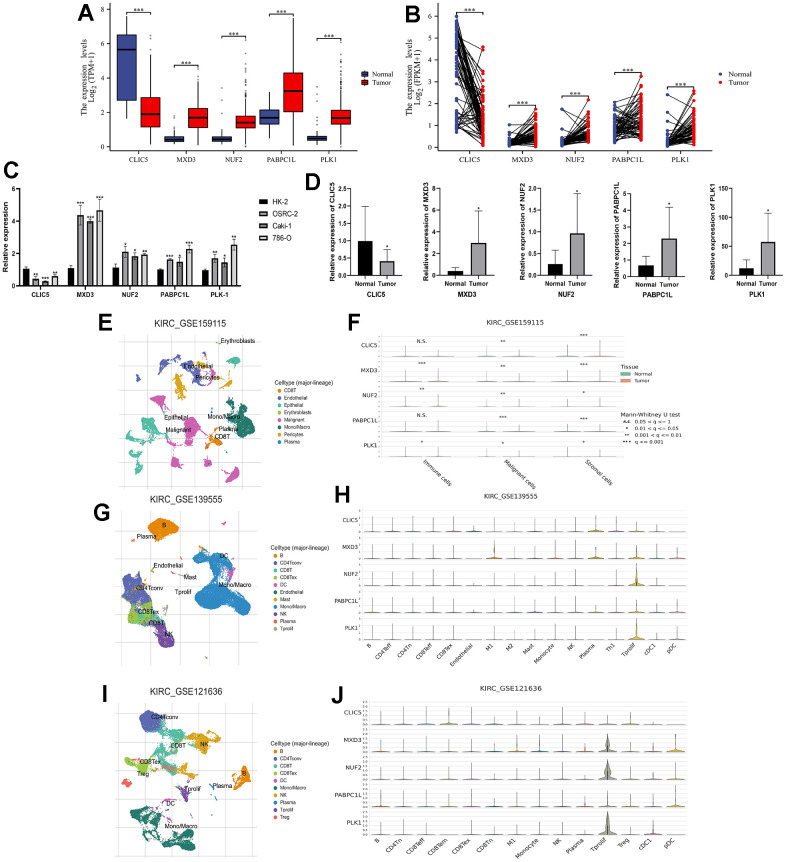
**Expression validation of key genes in risk signatures of LLPS-related genes and single-cell analysis.** (**A**) Unpaired expression analysis of the five key genes in the TCGA-KIRC cohort; (**B**) Paired expression analysis of the five key genes in the TCGA-KIRC cohort; (**C**) Expression of the five key genes in renal carcinoma cell lines (OSRC-2, Caki-1, and 786-O), with renal normal epithelial cells (HK-2) as a reference; (**D**) Key gene expression in ccRCC tissues and corresponding normal kidney tissues collected from our research centre; (**E**, **F**) GSE159115 cohort analysed the expression of five genes in immune cells, malignant cells and stromal cells; (**G**, **H**) GSE139555 analysed the expression of five genes in different cellular abundance; (**I**, **J**) GSE121636 further analysed the expression of five genes in different cells. (*p < 0.05, **p < 0.01, ***p < 0.001, “ns”: not statistically significant).

The new LLPS-associated gene risk model consists of five key genes (CLIC5, MXD3, NUF2, PABPC1L, PLK1). Therefore, we next performed qRT-PCR experiments to evaluate the mRNA expression of these genes in renal cancer cell lines and renal cancer tissues. The results of the TCGA-KIRC cohort showed that either in paired or unpaired renal cancer tissues, except for CLIC5, which was in a low expression state, the remaining four genes (MXD3, NUF2, PABPC1L, and PLK1) were in a high expression (Figure 8A, 8B). Normal renal tubular epithelial cells (Human Kidney-2, HK-2) were selected as a normal reference, and the findings in renal cancer cell lines (OSRC-2, Caki-1, 786-O) were also consistent with the results of the TCGA-KIRC cohort. Similarly, CLIC5 expression was found to be lower than that of normal kidney tissues in renal clear cell carcinoma tissues extracted from our research center, while MXD3, NUF2, PABPC1L, and PLK1 were significantly higher in renal clear cell carcinoma tissues than in normal kidney tissues (Figure 8C, 8D).

In order to further explore the five key genes, we also performed single-cell analysis to investigate their expression distribution in different cells. Analysis of the GSE159115 database showed that five genes in malignant and stromal cells differed significantly between tumour and normal tissues, but only MXD3, NUF2 and PLK1 in immune cells (Figure 8E, 8F). The GSE139555 and GSE121636 databases demonstrated the expression of the genes in a variety of cells. In GSE139555, except for CLIC5, which was more highly expressed in plasma, the rest of the genes were more highly expressed in Tprolif (Figure 8G, 8H). While in GSE121636 the results showed that CLIC5 was higher in CD8 Tem cells, PABPC1L had a higher percentage of expression in pDC, and the rest of the genes remained higher in Tprolif (Figure 8I, 8J).

## DISCUSSION

The mechanism of cancer development is full of complexity, and the discovery of LLPS has provided a new direction for its elucidation and treatment, which deserves more in-depth research. Various cellular activities involve “phase separation”, and therefore abnormal phase separation often leads to the development of many diseases. For example, in colorectal adenocarcinoma, SENP1 reduces the SUMOylation of RNF168 when DNA damage occurs, facilitating its LLPS, enhancing non-homologous end joining (NHEJ) repair efficiency, and consequently strengthening cancer cell resistance to DNA-damaging agents [26]. CircASH2 enhances the LLPS of nuclear Y-box binding protein 1 (YBX1) in hepatocellular carcinoma, and also enhances the attenuation of the primary target protein tropomyosin 4 (TPM4) transcripts, thereby altering the tumor cytoskeleton structure to inhibit HCC metastasis [27]. By promoting the interaction between the IL-6 enhancer and promoter, the phase separation of the YY1 complex in M2 macrophages triggers the upregulation of IL-6, further promoting the progression of PCa [28].

It has been shown that conventional TNM staging may be inaccurate in predicting the prognosis of ccRCC patients because it does not take into account the effect of gene expression on prognosis [29]. Many studies are currently trying to predict the prognosis of patients with ccRCC by establishing risk signatures based on the results of gene sequencing and combining them with clinical information [30–32]. But the overall efficiency of clinical translation is low. In this study, differential genes were selected based on differential expression from the many LLPS-related genes found. To prevent overfitting and select the genes with the most predictive value, LASSO Cox regression analysis screened five genes (CLIC5, MXD3, NUF2, PABPC1L, and PLK1) to construct a risk-prognostic prediction signature for LLPS-related genes in ccRCC. The signature distinguished significant differences in prognosis between the two groups of ccRCC patients in the TCGA-KIRC and E-MTAB-1980 cohorts, and had a more prominent ability to predict the prognosis and diagnosis of ccRCC patients. Notably, when the LLPS-related gene risk score was combined with age and tumour stage, the results of univariate and multivariate analyses indicated that the model was an independent prognostic factor for ccRCC. The risk signature with LLPS-related genes we constructed has a strong ability to judge the prognosis of ccRCC patients, which is a guide for clinicians’ diagnosis and treatment decisions. However, our study is still at the stage of public database analysis, which has certain limitations. If translated into practical clinical applications, more thorough evaluation and analysis in prospective, multicenter large data samples are needed.

The tumour microenvironment plays a crucial role in cancer development, especially in tumours with a high degree of immune infiltration ccRCC [33, 34]. The results of the immune infiltration analysis revealed a significantly higher infiltration abundance of Tregs and CD8+ T cells in the high-risk group than in the low-risk group. Tregs are lymphocytes that inhibit anti-tumour responses and the degree of infiltration of this cell was significantly correlated with poor prognosis [35, 36]. Several studies have shown that the finding of heavily infiltrating CD8+ T cells in ccRCC samples is strongly associated with poor prognosis [37–39]. The high expression of most of the genes analysed in single cells was positively correlated with Tprolif cells, which drive the expression of a number of markers associated with immune failure e.g., HAVCR2, CTLA4, LAG3, TIGIT and PDCD1) [40]. We further explored the relationship between immune checkpoint expression and high- and low-risk groups. As shown in the results, the expression levels of immune checkpoints were differential in different groups, especially PD-1, CTLA4 and LAG3, which are commonly found in renal cancer, were more significantly expressed in the high-risk group. Up-regulation of PD-1 and CTLA4 inhibits T-cell activation and proliferation, allowing tumours to evade immune recognition and elimination [41, 42]. LAG3 binds to MHC class II HLAs with high affinity, thereby promoting pro-inflammatory cytokine production and negatively regulating T cell responses [43]. This set of results could explain to some extent the higher immune cell infiltration as well as the higher expression of immune checkpoints accompanied by a poor prognosis in the portion of ccRCC patients in the high-risk group of LLPS-related gene risk scores.

The new LLPS-associated gene risk model is composed of CLIC5, MXD3, NUF2, PABPC1L, and PLK1, which have also been found to play a role in ccRCC in existing studies. Resistance to FOLFIRNOX in pancreatic ductal adenocarcinoma may result from MIR1307-induced reduction in CLIC5 expression [44]. Previous studies have shown the association of MXD3 expression levels and genetic or epigenetic alterations with tumour stage, metastasis, TME, immune escape and drug sensitivity in 39 TCGA cancer types and subtypes, including ccRCC [45]. Overexpression of NUF2 is associated with a poor prognosis in ccRCC and can act as a potential oncogene to promote proliferation, migration, and invasion of ccRCC cells by affecting the recruitment of KDM2A to modulate the H3K36me2 modification in the promoter region, which can activate the transcription of HMGA2 through epigenetics [46]. PABPC2L is highly expressed in colorectal cancer and significantly correlates with its clinical stage and prognosis [47]. PLK1 is highly expressed in ccRCC, is dependent on hypoxia/HIF-2 mediation, and is associated with poor prognosis and resistance to tyrosine kinase inhibitors of the VEGF receptor [48]. This provides a strong reference to further explore the role of these genes in regulating LLPS in ccRCC.

This study is the first to construct a prognostic risk model for ccRCC patients from the perspective of LLPS, and further analysed the potential function, immune infiltration and mutation, which can provide some help for clinicians’ therapeutic decision-making. However, our study still has some limitations. First, this study was based on a public database, which is a retrospective analysis with some limitations in the conclusions, and prospective studies are needed to validate these conclusions. Moreover, the public database itself has some limitations; we used only one cohort for validation, and the results obtained need to be validated with further clinical cohort data. Second, although risk signatures with LLPS-related genes performed well in predicting ccRCC survival, this study did not consider potential external factors, such as other important genes with predictive value, environmental factors, and lifestyle, which could have influenced the conclusions reached in this study. Finally, we only performed qRT-PCR experiments to validate the expression of key genes, lacking further biological evidence such as molecular function experiments.

## CONCLUSIONS

In conclusion, this study establishes a new risk model and column-line diagram for ccRCC patients from an LLPS perspective and can be used as a predictor of survival and prognosis for ccRCC patients and is expected to provide new solutions for clinical decision-making regarding immunotherapy and targeted therapy for ccRCC patients.

## MATERIALS AND METHODS

### Data source

RNA expression data of ccRCC patients and related clinical data were obtained from the TCGA-KIRC cohort on the TCGA website (https://portal.gdc.cancer.gov/), which contains 541 tumor samples and 72 normal samples. LLPS-related genes were obtained from the DrLLPS website (http://llps.biocuckoo.cn/index.php), which covers 887,164 known and computationally detected LLPS-associated proteins from 437 eukaryotic species [49]. We screened 3611 LLPS-related genes from the species “*Homo sapiens*” for subsequent analysis. RNA expression data and clinical data of 101 ccRCC patients from the E-MTAB-1980 cohort of the ArrayExpress database (https://www.ebi.ac.uk/biostudies/arrayexpress) were used as a validation cohort to test the validity of the signature.

### Screening for differentially expressed genes in ccRCC

The expression profiles of LLPS-related genes in the TCGA-KIRC cohort were extracted, and differentially expressed genes were screened using the “limma” package in R [50]. Filtering criteria required a p-value of <0.05 and |log2FC|> 1.

### Constructing and validating a prognostic signature for LLPS-related genes in ccRCC

Based on the results of differential expression analysis of LLPS-related genes in ccRCC, we used univariate Cox regression analysis to screen for prognostic genes by combining RNA expression data and clinical survival data. After that, the LASSO Cox regression analysis was performed using the “glmnet” package. Risk prediction signature was constructed by combining the screened genes and the calculated Coef’s coefficients.


$$\begin{array}{l} Risk\;Score=Exp_{RNA1}*Coef_{RNA1}+Exp_{RNA2}\\ \;\;\;\;\;\;\;\;\;\;\;\;\;\;\;\;\;\;\;*Coef_{RNA2}+\cdots +Exp_{RNAn}\\ \;\;\;\;\;\;\;\;\;\;\;\;\;\;\;\;\;\;\;*Coef_{RNAn} \end{array}$$

In the formula, “*Exp_RNAn_*” represented the expression value of a gene, and “*Coef_RNAn_*” represented the coefficient of the gene.

We performed a Kaplan-Meier curve analysis on the TCGA-KIRC and E-MATB-1980 cohorts, followed by a log-rank test to identify differences in OS between the two different cohorts. To assess the predictive power of the characteristics, we used ROC analysis. We meticulously organized pertinent clinical data and associated expression data pertaining to the high-risk and low-risk cohorts. Subsequently, we delved into the impact of other pertinent clinical variables on the prognostic efficacy of these risk-defined groups. Furthermore, we investigated the interrelationship between the risk scores and clinically relevant factors within the prognostic risk signature.

### Developing and verifying nomogram and calibration chart

Based on the univariate and multivariate Cox regression analyses, nomogram was constructed using the “rms” package, which included age, tumor stage, and risk group, and had predicted the 1-, 3-, and 5-year survival rates of patients with ccRCC. Calibration charts and ROC curves were further plotted to assess the reliability and accuracy of the plots.

### GO, KEGG and GESA analysis

The “limma” and “ClusterProfiler” packages calculated the differentially expressed genes in the high-risk and low-risk groups of the TCGA-KIRC cohort for GO and KEGG analysis in terms of BP, CC, and MF, respectively. For further revealing the biological functions of LLPS-related genes, we performed GSEA, and the annotation file “c2.all.v2023.1.Hs.symbols.gmt” was obtained from MsigD.

### Immune infiltration analysis and mutational landscape

The immune cell and immune-related pathway infiltration levels were calculated by the ssGESA algorithm and the “GSVA” and “GSEABase” packages aiming to assess the immune-related functions of patients with different LLPS-related gene risk scores. Secondly, the CIBERSORT algorithm was used to calculate the proportion of immune cell types infiltrated.

### Single-cell analysis of key genes for risk signature

Tumor Immune Single-cell Hub 2 (TISCH2, http://tisch.comp-genomics.org/home/) provides an online database for detailed single-cell analysis. We selected three data (GSE159115, GSE139555, and GSE121636) to analyze the distribution of five key genes (CLIC5, MXD3, NUF2, PABPC1L, and PLK1) in different cell types of ccRCC.

### ccRCC cell lines and tissues validate RNA expression of key genes

All cells were purchased from the Chinese Academy of Sciences (Shanghai, China). Cells were cultured in high-glucose DMEM (Solarbio, Beijing Solarbio Science and Technology Co., Ltd., Beijing, China), MEM (Boster, China) and 1,640 (Gibco, Carlsbad, CA, USA) medium containing 10% FBS (VivaCell Biosciences Co., Ltd., Shanghai, China) and 1% streptomycinpenicillin, and maintained at 37° C and 5% CO_2_.

Total RNA was extracted from three renal cancer cell lines (OSRC-2, Caki-1, 786-O) and HK-2, as well as from eight pairs of human renal cancer tissues and adjacent normal renal tissues using the TRIzol reagent (Cwbio, Jiangsu, China). Reverse transcription to cDNA was done under the instructions of the Reverse Transcription Kit (TransGen Biotech, China, Beijing). Finally, qPCR was performed using SYBR Real-Time PCR Kit (TransGen Biotech, China, Beijing). β-actin was used as an internal reference, and primer sequences for the remaining molecules are shown in Supplementary Table 1.

### Statistical analysis

Statistical analysis for this study was performed using R software (version 4.3.1) and GraphPad Prism (version 9.0). For comparison of differences between binary and continuous variables we used the student’s t-test. p < 0.05 was considered statistically significant.

## Supplementary Material

Supplementary Table 1

Supplementary Table 2

## References

[1] Siegel RL, Miller KD, Wagle NS, Jemal A. Cancer statistics, 2023. CA Cancer J Clin. 2023; 73:17–48. 10.3322/caac.21763 36633525

[2] Zhang D, Ni Y, Wang Y, Feng J, Zhuang N, Li J, Liu L, Shen W, Zheng J, Zheng W, Qian C, Shan J, Zhou Z. Spatial heterogeneity of tumor microenvironment influences the prognosis of clear cell renal cell carcinoma. J Transl Med. 2023; 21:489. 10.1186/s12967-023-04336-8 37474942 PMC10360235

[3] Jonasch E, Gao J, Rathmell WK. Renal cell carcinoma. BMJ. 2014; 349:g4797. 10.1136/bmj.g4797 25385470 PMC4707715

[4] Makhov P, Joshi S, Ghatalia P, Kutikov A, Uzzo RG, Kolenko VM. Resistance to Systemic Therapies in Clear Cell Renal Cell Carcinoma: Mechanisms and Management Strategies. Mol Cancer Ther. 2018; 17:1355–64. 10.1158/1535-7163.MCT-17-1299 29967214 PMC6034114

[5] Qu Y, Feng J, Wu X, Bai L, Xu W, Zhu L, Liu Y, Xu F, Zhang X, Yang G, Lv J, Chen X, Shi GH, et al. A proteogenomic analysis of clear cell renal cell carcinoma in a Chinese population. Nat Commun. 2022; 13:2052. 10.1038/s41467-022-29577-x 35440542 PMC9019091

[6] D’Aniello C, Berretta M, Cavaliere C, Rossetti S, Facchini BA, Iovane G, Mollo G, Capasso M, Pepa CD, Pesce L, D’Errico D, Buonerba C, Di Lorenzo G, et al. Biomarkers of Prognosis and Efficacy of Anti-angiogenic Therapy in Metastatic Clear Cell Renal Cancer. Front Oncol. 2019; 9:1400. 10.3389/fonc.2019.01400 31921657 PMC6917607

[7] Barata PC, Rini BI. Treatment of renal cell carcinoma: Current status and future directions. CA Cancer J Clin. 2017; 67:507–24. 10.3322/caac.21411 28961310

[8] Senturk A, Sahin AT, Armutlu A, Kiremit MC, Acar O, Erdem S, Bagbudar S, Esen T, Tuncbag N, Ozlu N. Quantitative Proteomics Identifies Secreted Diagnostic Biomarkers as well as Tumor-Dependent Prognostic Targets for Clear Cell Renal Cell Carcinoma. Mol Cancer Res. 2021; 19:1322–37. 10.1158/1541-7786.MCR-21-0004 33975903

[9] Corrò C, Moch H. Biomarker discovery for renal cancer stem cells. J Pathol Clin Res. 2018; 4:3–18. 10.1002/cjp2.91 29416873 PMC5783955

[10] Marchioni M, Kriegmair M, Heck M, Amiel T, Porpiglia F, Ceccucci E, Campi R, Minervini A, Mari A, Van Bruwaene S, Linares E, Hevia V, Musquera M, et al, and EAU-Young Academic Urologist Kidney Cancer Group. Development of a Novel Risk Score to Select the Optimal Candidate for Cytoreductive Nephrectomy Among Patients with Metastatic Renal Cell Carcinoma. Results from a Multi-institutional Registry (REMARCC). Eur Urol Oncol. 2021; 4:256–63. 10.1016/j.euo.2020.12.010 33384274

[11] Mirza-Aghazadeh-Attari M, Mohammadzadeh A, Yousefi B, Mihanfar A, Karimian A, Majidinia M. 53BP1: A key player of DNA damage response with critical functions in cancer. DNA Repair (Amst). 2019; 73:110–9. 10.1016/j.dnarep.2018.11.008 30497961

[12] Taniue K, Akimitsu N. Aberrant phase separation and cancer. FEBS J. 2022; 289:17–39. 10.1111/febs.15765 33583140

[13] Zhang JZ, Mehta S, Zhang J. Liquid-liquid phase separation: a principal organizer of the cell’s biochemical activity architecture. Trends Pharmacol Sci. 2021; 42:845–56. 10.1016/j.tips.2021.07.003 34373114 PMC8858030

[14] Boeynaems S, Alberti S, Fawzi NL, Mittag T, Polymenidou M, Rousseau F, Schymkowitz J, Shorter J, Wolozin B, Van Den Bosch L, Tompa P, Fuxreiter M. Protein Phase Separation: A New Phase in Cell Biology. Trends Cell Biol. 2018; 28:420–35. 10.1016/j.tcb.2018.02.004 29602697 PMC6034118

[15] Decker CJ, Parker R. P-bodies and stress granules: possible roles in the control of translation and mRNA degradation. Cold Spring Harb Perspect Biol. 2012; 4:a012286. 10.1101/cshperspect.a012286 22763747 PMC3428773

[16] Chong PA, Vernon RM, Forman-Kay JD. RGG/RG Motif Regions in RNA Binding and Phase Separation. J Mol Biol. 2018; 430:4650–65. 10.1016/j.jmb.2018.06.014 29913160

[17] Wan L, Chong S, Xuan F, Liang A, Cui X, Gates L, Carroll TS, Li Y, Feng L, Chen G, Wang SP, Ortiz MV, Daley SK, et al. Impaired cell fate through gain-of-function mutations in a chromatin reader. Nature. 2020; 577:121–6. 10.1038/s41586-019-1842-7 31853060 PMC7061414

[18] Wu Y, Zhou L, Zou Y, Zhang Y, Zhang M, Xu L, Zheng L, He W, Yu K, Li T, Zhang X, Chen Z, Zhang R, et al. Disrupting the phase separation of KAT8-IRF1 diminishes PD-L1 expression and promotes antitumor immunity. Nat Cancer. 2023; 4:382–400. 10.1038/s43018-023-00522-1 36894639 PMC10042735

[19] Miete C, Solis GP, Koval A, Brückner M, Katanaev VL, Behrens J, Bernkopf DB. Gαi2-induced conductin/axin2 condensates inhibit Wnt/β-catenin signaling and suppress cancer growth. Nat Commun. 2022; 13:674. 10.1038/s41467-022-28286-9 35115535 PMC8814139

[20] Bouchard JJ, Otero JH, Scott DC, Szulc E, Martin EW, Sabri N, Granata D, Marzahn MR, Lindorff-Larsen K, Salvatella X, Schulman BA, Mittag T. Cancer Mutations of the Tumor Suppressor SPOP Disrupt the Formation of Active, Phase-Separated Compartments. Mol Cell. 2018; 72:19–36.e8. 10.1016/j.molcel.2018.08.027 30244836 PMC6179159

[21] You Q, Chen JY, Wu XH, Xue YT, Sun JB, Wei Y, Zheng QS, Xue XY, Chen DN, Xu N. A Liquid-Liquid Phase Separation-Related Index Associate with Biochemical Recurrence and Tumor Immune Environment of Prostate Cancer Patients. Int J Mol Sci. 2023; 24:5515. 10.3390/ijms24065515 36982591 PMC10058551

[22] Wang J, Meng F, Mao F. Single cell sequencing analysis and transcriptome analysis constructed the liquid-liquid phase separation(LLPS)-related prognostic model for endometrial cancer. Front Oncol. 2022; 12:1005472. 10.3389/fonc.2022.1005472 36185238 PMC9515536

[23] Yu-Qing H, Peng-Ping L, Ke S, Ke-Xing Y, Wei-Jun Z, Zhen-Yu W. Comprehensive analysis of liquid-liquid phase separation-related genes in prediction of breast cancer prognosis. Front Genet. 2022; 13:834471. 10.3389/fgene.2022.834471 36246644 PMC9554098

[24] Fang ZS, Zhang Z, Liang ZJ, Long ZR, Xiao Y, Liang ZY, Sun X, Li HM, Huang H. Liquid-Liquid Phase Separation-Related Genes Associated with Tumor Grade and Prognosis in Hepatocellular Carcinoma: A Bioinformatic Study. Int J Gen Med. 2021; 14:9671–9. 10.2147/IJGM.S342602 34934344 PMC8684409

[25] Qiu Y, Pan M, Chen X. A Liquid-Liquid Phase Separation-Related Gene Signature as Prognostic Biomarker for Epithelial Ovarian Cancer. Front Oncol. 2021; 11:671892. 10.3389/fonc.2021.671892 34168991 PMC8217755

[26] Wei M, Huang X, Liao L, Tian Y, Zheng X. SENP1 Decreases RNF168 Phase Separation to Promote DNA Damage Repair and Drug Resistance in Colon Cancer. Cancer Res. 2023; 83:2908–23. 10.1158/0008-5472.CAN-22-4017 37350666

[27] Liu B, Shen H, He J, Jin B, Tian Y, Li W, Hou L, Zhao W, Nan J, Zhao J, Shen J, Yu H, Wang Y, et al. Cytoskeleton remodeling mediated by circRNA-YBX1 phase separation suppresses the metastasis of liver cancer. Proc Natl Acad Sci USA. 2023; 120:e2220296120. 10.1073/pnas.2220296120 37459535 PMC10372620

[28] Chen S, Lu K, Hou Y, You Z, Shu C, Wei X, Wu T, Shi N, Zhang G, Wu J, Chen S, Zhang L, Li W, et al. YY1 complex in M2 macrophage promotes prostate cancer progression by upregulating IL-6. J Immunother Cancer. 2023; 11:e006020. 10.1136/jitc-2022-006020 37094986 PMC10152059

[29] Motzer RJ, Jonasch E, Michaelson MD, Nandagopal L, Gore JL, George S, Alva A, Haas N, Harrison MR, Plimack ER, Sosman J, Agarwal N, Bhayani S, et al, and CGC. NCCN Guidelines Insights: Kidney Cancer, Version 2.2020. J Natl Compr Canc Netw. 2019; 17:1278–85. 10.6004/jnccn.2019.0054 31693980

[30] Chen J, Jiang R, Guan W, Cao Q, Tian Y, Dong K, Pan X, Cui X. Novel model of pyroptosis-related molecular signatures for prognosis prediction of clear cell renal cell carcinoma patients. Int J Med Sci. 2024; 21:496–507. 10.7150/ijms.88301 38250606 PMC10797671

[31] Wu Q, Sun Y, Qin X, Li M, Huang S, Wang X, Weng G. Development and validation of a novel anoikis-related gene signature in clear cell renal cell carcinoma. Front Oncol. 2023; 13:1211103. 10.3389/fonc.2023.1211103 37965453 PMC10641395

[32] Liu G, Li F, Ge Y, Shi Y, Ren F, Zhu L. Prognosis, Immune Microenvironment Infiltration and Immunotherapy Response in Clear Cell Renal Cell Carcinoma Based on Cuproptosis-related Immune Checkpoint Gene Signature. J Cancer. 2023; 14:3335–50. 10.7150/jca.88467 37928426 PMC10622984

[33] Vuong L, Kotecha RR, Voss MH, Hakimi AA. Tumor Microenvironment Dynamics in Clear-Cell Renal Cell Carcinoma. Cancer Discov. 2019; 9:1349–57. 10.1158/2159-8290.CD-19-0499 31527133 PMC6774890

[34] Şenbabaoğlu Y, Gejman RS, Winer AG, Liu M, Van Allen EM, de Velasco G, Miao D, Ostrovnaya I, Drill E, Luna A, Weinhold N, Lee W, Manley BJ, et al. Tumor immune microenvironment characterization in clear cell renal cell carcinoma identifies prognostic and immunotherapeutically relevant messenger RNA signatures. Genome Biol. 2016; 17:231. 10.1186/s13059-016-1092-z 27855702 PMC5114739

[35] Dees S, Ganesan R, Singh S, Grewal IS. Regulatory T cell targeting in cancer: Emerging strategies in immunotherapy. Eur J Immunol. 2021; 51:280–91. 10.1002/eji.202048992 33302322

[36] Liotta F, Gacci M, Frosali F, Querci V, Vittori G, Lapini A, Santarlasci V, Serni S, Cosmi L, Maggi L, Angeli R, Mazzinghi B, Romagnani P, et al. Frequency of regulatory T cells in peripheral blood and in tumour-infiltrating lymphocytes correlates with poor prognosis in renal cell carcinoma. BJU Int. 2011; 107:1500–6. 10.1111/j.1464-410X.2010.09555.x 20735382

[37] Dai S, Zeng H, Liu Z, Jin K, Jiang W, Wang Z, Lin Z, Xiong Y, Wang J, Chang Y, Bai Q, Xia Y, Liu L. Intratumoral CXCL13+CD8+T cell infiltration determines poor clinical outcomes and immunoevasive contexture in patients with clear cell renal cell carcinoma. J Immunother Cancer. 2021; 9:e001823. 10.1136/jitc-2020-001823 33589528 PMC7887366

[38] Koh MY, Sayegh N, Agarwal N. Seeing the forest for the trees-single-cell atlases link CD8+ T cells and macrophages to disease progression and treatment response in kidney cancer. Cancer Cell. 2021; 39:594–6. 10.1016/j.ccell.2021.03.008 33861995

[39] Ghatalia P, Gordetsky J, Kuo F, Dulaimi E, Cai KQ, Devarajan K, Bae S, Naik G, Chan TA, Uzzo R, Hakimi AA, Sonpavde G, Plimack E. Prognostic impact of immune gene expression signature and tumor infiltrating immune cells in localized clear cell renal cell carcinoma. J Immunother Cancer. 2019; 7:139. 10.1186/s40425-019-0621-1 31138299 PMC6540413

[40] Yang C, Guo Y, Wu Z, Huang J, Xiang B. Comprehensive Analysis of Cuproptosis-Related Genes in Prognosis and Immune Infiltration of Hepatocellular Carcinoma Based on Bulk and Single-Cell RNA Sequencing Data. Cancers (Basel). 2022; 14:5713. 10.3390/cancers14225713 36428805 PMC9688556

[41] Monjaras-Avila CU, Lorenzo-Leal AC, Luque-Badillo AC, D’Costa N, Chavez-Muñoz C, Bach H. The Tumor Immune Microenvironment in Clear Cell Renal Cell Carcinoma. Int J Mol Sci. 2023; 24:7946. 10.3390/ijms24097946 37175653 PMC10178526

[42] Pardoll DM. The blockade of immune checkpoints in cancer immunotherapy. Nat Rev Cancer. 2012; 12:252–64. 10.1038/nrc3239 22437870 PMC4856023

[43] Giraldo NA, Becht E, Pagès F, Skliris G, Verkarre V, Vano Y, Mejean A, Saint-Aubert N, Lacroix L, Natario I, Lupo A, Alifano M, Damotte D, et al. Orchestration and Prognostic Significance of Immune Checkpoints in the Microenvironment of Primary and Metastatic Renal Cell Cancer. Clin Cancer Res. 2015; 21:3031–40. 10.1158/1078-0432.CCR-14-2926 25688160

[44] Carotenuto P, Amato F, Lampis A, Rae C, Hedayat S, Previdi MC, Zito D, Raj M, Guzzardo V, Sclafani F, Lanese A, Parisi C, Vicentini C, et al. Modulation of pancreatic cancer cell sensitivity to FOLFIRINOX through microRNA-mediated regulation of DNA damage. Nat Commun. 2021; 12:6738. 10.1038/s41467-021-27099-6 34795259 PMC8602334

[45] Wu SY, Lin KC, Lawal B, Wu ATH, Wu CZ. MXD3 as an onco-immunological biomarker encompassing the tumor microenvironment, disease staging, prognoses, and therapeutic responses in multiple cancer types. Comput Struct Biotechnol J. 2021; 19:4970–83. 10.1016/j.csbj.2021.08.047 34584637 PMC8441106

[46] Lin J, Chen X, Yu H, Min S, Chen Y, Li Z, Xie X. NUF2 Drives Clear Cell Renal Cell Carcinoma by Activating HMGA2 Transcription through KDM2A-mediated H3K36me2 Demethylation. Int J Biol Sci. 2022; 18:3621–35. 10.7150/ijbs.70972 35813477 PMC9254462

[47] Wu YQ, Ju CL, Wang BJ, Wang RG. PABPC1L depletion inhibits proliferation and migration via blockage of AKT pathway in human colorectal cancer cells. Oncol Lett. 2019; 17:3439–45. 10.3892/ol.2019.9999 30867782 PMC6396114

[48] Dufies M, Verbiest A, Cooley LS, Ndiaye PD, He X, Nottet N, Souleyreau W, Hagege A, Torrino S, Parola J, Giuliano S, Borchiellini D, Schiappa R, et al. Plk1, upregulated by HIF-2, mediates metastasis and drug resistance of clear cell renal cell carcinoma. Commun Biol. 2021; 4:166. 10.1038/s42003-021-01653-w 33547392 PMC7865059

[49] Ning W, Guo Y, Lin S, Mei B, Wu Y, Jiang P, Tan X, Zhang W, Chen G, Peng D, Chu L, Xue Y. DrLLPS: a data resource of liquid-liquid phase separation in eukaryotes. Nucleic Acids Res. 2020; 48:D288–95. 10.1093/nar/gkz1027 31691822 PMC7145660

[50] Ritchie ME, Phipson B, Wu D, Hu Y, Law CW, Shi W, Smyth GK. limma powers differential expression analyses for RNA-sequencing and microarray studies. Nucleic Acids Res. 2015; 43:e47. 10.1093/nar/gkv007 25605792 PMC4402510

