# Supplementary material for: A novel risk signature based on liquid-liquid phase separation-related genes reveals prognostic and tumour microenvironmental features in clear cell renal cell carcinoma
Source: Aging (Albany NY). 2024 Mar 27;16(7):6118–34. doi: 10.18632/aging.205691 (PMC11042959; doi:10.18632/aging.205691)
Supplement: Supplementary Table 1 [file aging-16-205691-s001.pdf]

SUPPLEMENTARY TABLES

Supplementary Table 1. Primer sequences for related genes.

	Forward sequence(5'-3')	Reverse sequence(5'-3')
NUF2	TGGAGACTCAGTTGACTGCCTG	ATTTGGTCCTCCAAGTTCAGGCT
PLK1	TACGGCAAATTGTGCTTGGC	CCCACACAGGGTCTTCTTCC
MXD3	GAGCATGGTTATGCGTCCCTGT	CCTGCGCTTCTCCAGTTCATTG
PABPC1L	CGTCATCAATGGAGTCGTCCAG	AGCGGCAGAATGAACTGAAGCG
CLIC5	TCTGTTGCCCAAGCTCCATGTG	GCATAGGCGTTCTTGAGGTACC
β-actin	TCTCCCAAGTCCACACAGG	GGCACGAAGGCTCATCA
